# Epigenome Editing of Potato by Grafting Using Transgenic Tobacco as siRNA Donor

**DOI:** 10.1371/journal.pone.0161729

**Published:** 2016-08-26

**Authors:** Atsushi Kasai, Songling Bai, Hatsune Hojo, Takeo Harada

**Affiliations:** 1 Department of Agriculture and Life Science, Hirosaki University, Hirosaki, Japan; 2 College of Agriculture & Biotechnology, Zhejiang University, Hangzhou, Zhejiang Province, China; Inc, UNITED STATES

## Abstract

In plants, it is possible to induce heritable transcriptional gene silencing (TGS) via RNA-directed DNA methylation (RdDM) using artificially synthesized small RNA (siRNA) homologous to the 5'-flanking region of the target gene. As the siRNA signal with a specific RNA determinant moves through plasmodesmata and sieve elements, we attempted to induce TGS of a transgene and an endogenous gene of potato (*Solanum tuberosum*) rootstock by grafting using siRNA produced in a tobacco (*Nicotiana benthamiana*) scion. Our results provide evidence that this system can induce TGS of target genes in tubers formed on potato rootstock. The TGS is maintained in the progeny tubers lacking the transported siRNAs. Our findings reveal that epigenome editing using mobile RNA has the potential to allow breeding of artificial sport cultivars in vegetative propagation crops.

## Introduction

The term epigenetics generally refers to the study of heritable information that is independent of DNA sequence variation. Epigenetic changes occur at the DNA level through DNA methylation of cytosine residues or at the level of histones that influence the accessibility of the DNA to transcription activation [1]. A number of studies have revealed that siRNA initiates gene silencing through the RNA-directed DNA methylation (RdDM) pathway [2, 3]. Indeed, a small RNA (siRNA) homologous to the promoter sequence of a gene can cause transcriptional gene silencing (TGS) of that gene [4]. Epigenetic change is an important mechanism for regulation of genomic integrity in higher eukaryotes. Since this information functions as a transcriptional memory associated with cell fate decisions, developmental switches, or stress responses, the memory needs to be erased during reproduction, as is the case for the vernalization memory in wheat [5, 6]. By contrast, some reports have indicated that transgenerational epigenetic memory can be stably transmitted through meiosis, resulting in inheritance by the subsequent generation [7–10]. Therefore, artificial creation of novel epi-alleles is a promising approach for improvement of crops [11, 12]. Nonetheless, a major concern regarding epimutants is that they may be partially heritable through gametogenesis. On the other hand, a number of commonly cultivated plants, such as potato, sugar cane, and fruit trees, are usually propagated by vegetative means rather than by seeds. Therefore, TGS would be a particularly attractive approach for improvement of such clonally propagated species. Several studies have demonstrated that transgene-derived siRNA moves across the graft union between a scion and a rootstock [13–15]. We have also achieved stable TGS status of a transgene in *Nicotiana benthamiana* by graft-transported siRNAs derived from the hairpin RNA of the promoter sequence [16]. Here, we attempted to induce TGS in potato tuber through hetero-grafting using tobacco as a siRNA donor.

## Results and Discussion

**Fig 1:**
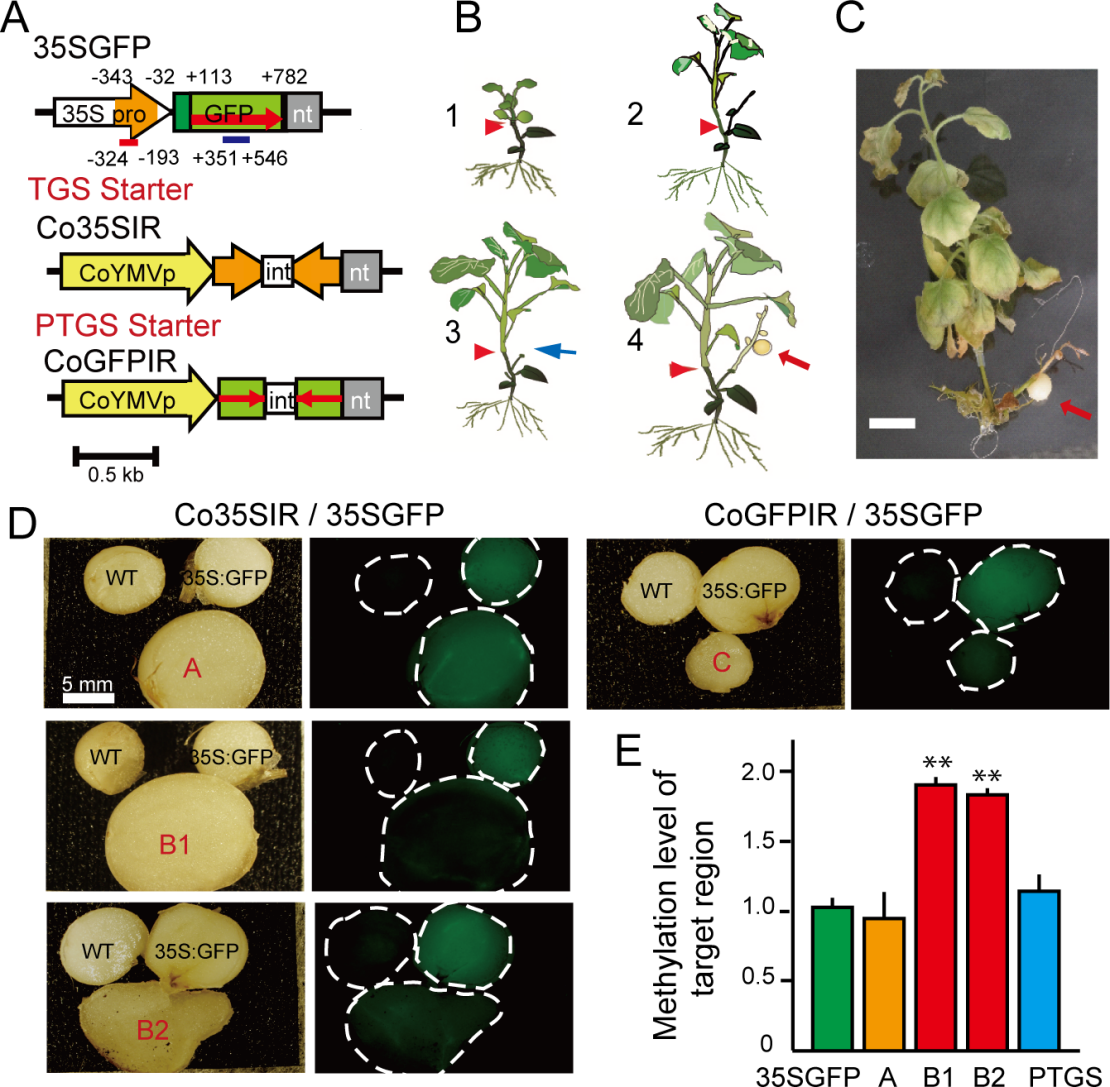
TGS of 35S:GFP transgene in micro tubers formed on hetero-grafted plants. (**A**) Schematic diagram of the silencing starters. The TGS starter (35SpIR) contained an inverted repeat (IR) of the 35S promoter sequence (-32 ~ -343). The PTGS starter contained an IR of part of GFP (+113 ~ +782). CoYMV is a companion cell-specific promoter. (**B**) Schematic drawing of grafting and micro-tuber (MT) formation on the grafted plant. The MT (arrow) was generated spontaneously from a callus formed on the cut surface of a lateral potato shoot. Red arrowhead shows the graft union. (**C**) MT formed on a grafted plant between the *N*. *benthamiana* scion and the potato stock. (**D**) GFP expression and silencing of MTs. The MTs were cut with a razor and the surface was observed under UV light. (**E**) Levels of methylation of the target region. Asterisks show statistically significant (**; P<0.01 Student's t test) differences relative to 35SGFP. Means and SD of 2 to 4 biological replicates are shown.

**Fig 2:**
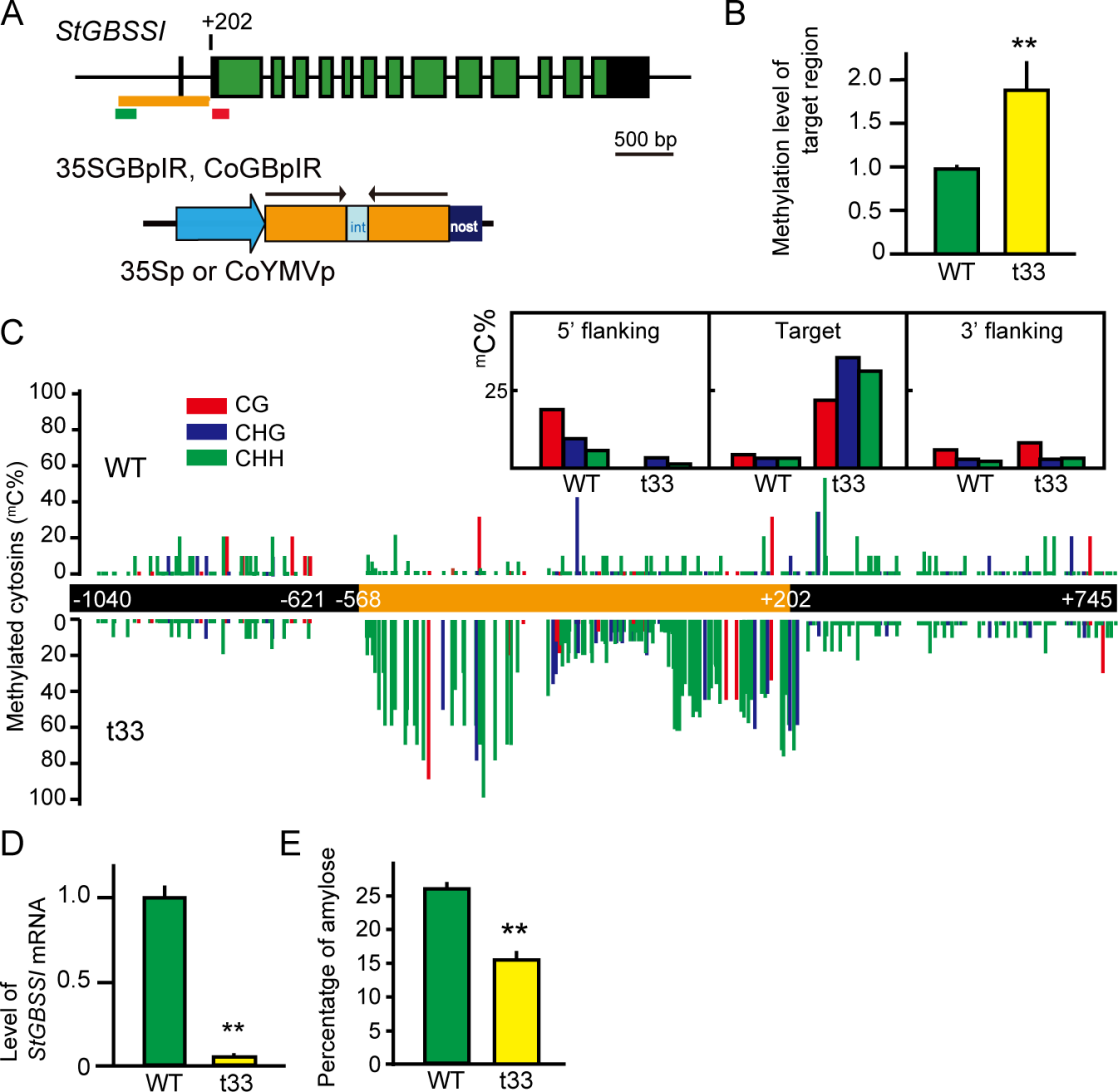
TGS induction of the endogenous gene *StGBSSI* in 35SGBpIR transgenic potato. (**A**) Schematic diagram of the *GBSSI* 5'-flanking region in potato ‘Waseshiro’. Black boxes are untranslated regions, and green boxes are exons. Orange bar shows the target region. Green and red bars indicate regions used for methylation level and transcription level analyses, respectively. The construct of 35SGBpIR and CoGBpIR is drawn schematically. (**B**) Methylation level of the target region in the potato leaves of *in vitro* sub-culturing shoots. WT: wild type, t33: 35SGBpIR line 33. Asterisks show statistically significant (**; P<0.01 Student's t test) differences relative to 35SGFP. Means and SD of 3 biological replicates are shown. (**C**) DNA methylation status in the target (orange) and its flanking regions (black) of *GBSSI*. The leaves were subjected to bisulphite sequencing. The percentages of methylation at individual cytosines are shown. Upper and lower are WT and t33 results, respectively. 5'-flanking region from -621 to -568 couldn't analyze due to lack of appropriate primer sets. The methylation rates of cytosines with different sequence backgrounds are shown in the inset box. (**D**) Down-regulation of the *GBSSI* transcript level in t33. (**E**) Percentage of amylose in the starch of WT and t33 tubers (n = 5) which were grown in a glasshouse.

**Fig 3:**
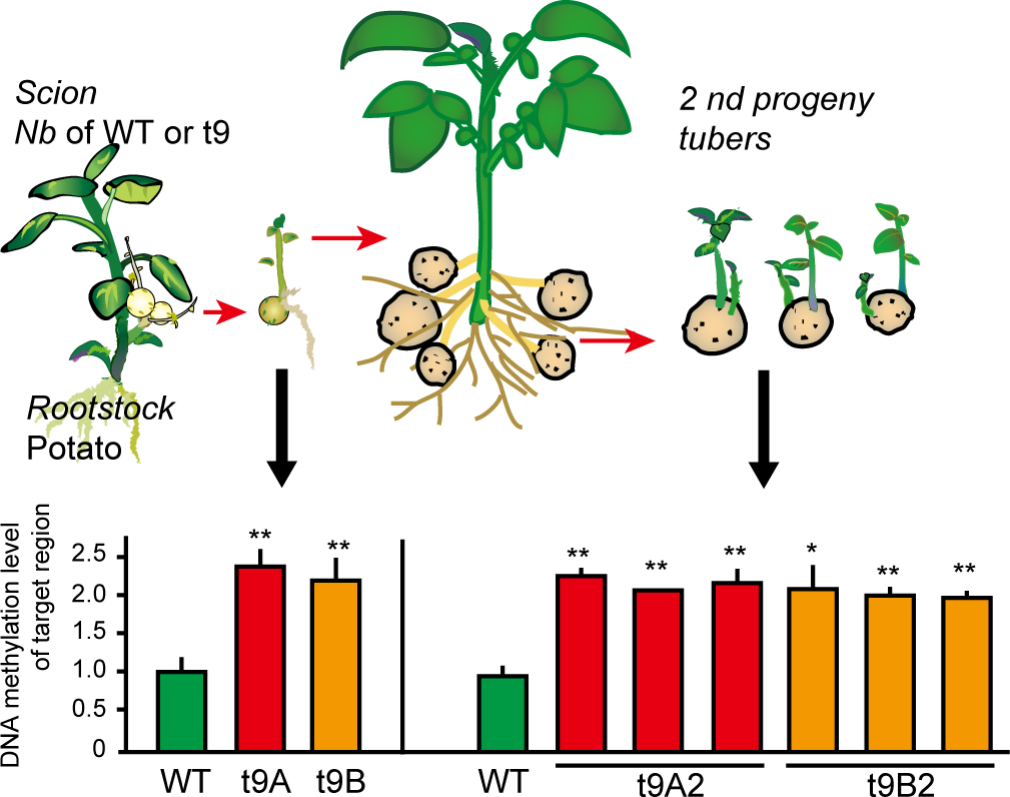
DNA methylation in the MTs formed trans-grafting plant and its stability in the progenies. Two MTs formed on a grafted plant and their progeny tubers were analyzed. Actually, the sprouts from tubers were used as analyzing materials. Asterisks show statistically significant (*; p<0.05, **; P<0.01 Student's t test) differences relative to WT. Means and SD of 3 biological replicates are shown.

**Fig 4:**
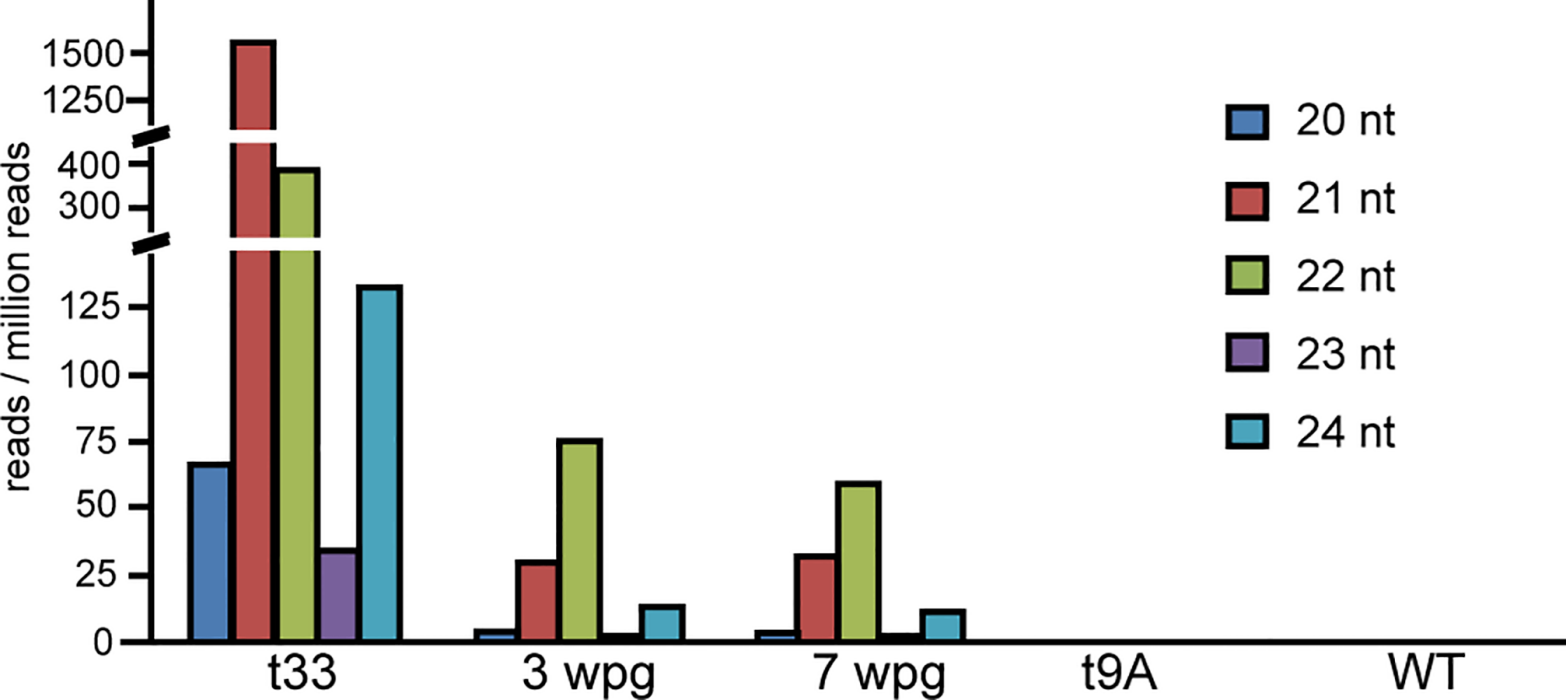
Fractions of 20–24 nt siRNA mapped to *GBSSI* target region in different materials. 3 wag and 7 wag are adventitious shoots (Fig 1B4) in rootstock of trans-grafted plants at 3 weeks and 7 weeks after grafting, respectively. Epi-A is t9A in Fig 3.

First, the promoter of the 35S:GFP locus in transgenic potato was targeted by siRNA derived from the TGS starter (Co35SpIR, Fig 1A) to induce trans-locational TGS [16]. In order to enhance the potential siRNA level in the phloem, the transcription of Co35SpIR was controlled by a companion cell-specific promoter (Commelina Yellow Mottle Virus promoter) [17]. A post-transcriptional gene silencing (PTGS) starter (CoGFPIR) for the 35S:GFP transgene was also constructed for comparison with the TGS. The Co35SpIR transgenic *N*. *benthamiana* with confirmed siRNA production ability was then grafted onto the 35S:GFP potato rootstock (Fig 1B-1). The hetero-grafted plants were grown aseptically in a tissue culture vessel. Immediately after grafting, the shoot(s) from lateral bud(s) in the potato stock sprouted and grew actively (Fig 1B-2) because of transient loss of apical dominance until secure establishment of the graft union. Meanwhile, the graft union showed strict conglutination, resulting in active growth of the scion. After approximately one month, the grown lateral shoot of the potato rootstock was cut off to increase the sink power for phloem transport of siRNA into the rootstock organ (Fig 1B-3; blue arrow). Unexpectedly, in approximately 20% of cases, an adventitious shoot was regenerated spontaneously from the compact callus formed on the cut surface of the lateral shoot (Fig 1B-4 and 1C and S1 Fig). The resulting plants were then placed on micro-tuber (MT) induction medium [18].

One and two MTs (Fig 1B and 1C) were formed on two grafted plants, respectively. Although one tuber from grafted plant A exhibited almost the same GFP expression as the 35S:GFP control, two MTs (tubers B1 and B2) from grafted plant B showed decreased expression (Fig 1D). Furthermore, they showed clearly high methylation of the target region (Fig 1E and S2A Fig). In the case of PTGS, a decrease of the GFP transcript was observed, but the methylation level was unchanged (Fig 1E). These methylation levels in the target region were maintained in the shoots that had sprouted from the MTs (S2A Fig). Furthermore, the 2nd progeny MTs on the sprouted shoots derived from tuber B1 exhibited low GFP expression and a low GFP mRNA level, whereas the PTGS case no longer showed the silencing status (S2B and S2C Fig). A second round of experiments showed that one out of four MTs of hetero-grafted plants exhibited clear TGS, which was also supported by the high methylation level of only the target region (S3 Fig).

It has been shown that siRNA transported in phloem from the scion induces strong TGS in lateral roots including root apical meristem cells in the rootstock [16], because in angiosperms lateral roots are initiated from only two pericycle cells [19, 20], in which TGS is induced by cell to cell movement of siRNA from sieve elements to phloem cells. Indeed, potato lateral roots harvested from the hetero-grafted plants manifested clear TGS as a result of low levels of GFP expression and hypermethylation of the target region (S4 Fig). Furthermore, the shoot regenerated from the root and the MT on the shoot also exhibited the TGS state (S5 Fig). These results indicated that derivation of a shoot from a TGS lateral root by tissue culture is an infallible method of obtaining a TGS potato plant, despite the long period (approximately 6 months) needed to obtain a sufficiently strong shoot.

Next, the TGS induction system using graft-transported siRNA was applied to an endogenous gene in potato. Granule-bound starch synthase I (GBSSI) catalyses the synthesis of amylose in amyloplasts [21]. When the *GBSSI* was silenced by PTGS or TGS using siRNA, low-amylose and high-amylopectin potato starch was produced in the transgenic potato [22, 23]. The resulting waxy-type potato starch has a smooth pulpy texture, a high-quality taste with high viscosity, and is less retrograde in comparison to regular potato starch. In spite of its high commercial value, this type of improved potato is not cultivated because of the general public distrust of gene-modified (GM) crops [24]. Therefore, using graft-induced epigenetic change, we tried to create a TGS potato for *GBSSI* lacking the transgene as the TGS starter. The cultivar ‘Waseshiro’ has two allelic promoter sequences of *GBSSI*, which possesses 3 SNPs and four indel sequences between them (S6 Fig). A transgenic potato harboring 35S:GBpIR (line t33, Fig 2A and S7 Fig), which was constructed from the allele b sequence (S6 Fig) in accordance with a previous study [21], exhibited methylation of the genome target region (Fig 2B and 2C) and a reduced level of *GBSSI* mRNA (Fig 2D). The tubers harvested from pot cultivation had lower-amylose starch, with approximately 41% less amylose than the wild-type potato (Fig 2E), indicating that the siRNA starter we had designed was fully functional. The CoGBpIR *N*. *benthamiana* producing siRNA for the 5' flanking region of *GBSSI* (allele a, Fig 2A and S6 Fig) was generated, and then the siRNA production ability was confirmed by northern blotting (S8 Fig). These were grafted as a scion onto potato cultivar ‘Waseshiro’ shoots and grown in tissue culture bottles. Fifteen hetero-grafted plants produced MTs on the adventitious shoots (Fig 1B). One of them, the t9 *N*. *benthamiana* line (S8 Fig), was used as the scion, formed two MTs exhibiting a high methylation level in the target region, and the methylation status was maintained in the sprouted shoots of the 2nd progeny tubers (Fig 3). The methylation level was almost the same as that of the 35S:GBpIR transgenic potato exhibiting a low amylose-starch ratio compared with the wild-type ‘Waseshiro’. Therefore, we succeeded in generating a TGS potato line for the *GBSSI* gene. As a matter of course, these TGS potato plants had no fragments corresponding the TGS starter construct in the genome (S9 Fig). The shoot regenerated from the root also exhibited the appropriate *GBSSI* methylation levels (S10 Fig). We plan to analyze the relationship between methylation levels and TGS using these *GBSSI* TGS lines.

To clarify whether the siRNAs transported via the graft union remain in the epigenetically changed potato, we sequenced and analyzed small RNA pools derived from the respective materials. The siRNA profile of 35SGBpIR transgenic potato (t33) showed 2169 reads/million sequence reads (reads/MR) of the target-specific siRNAs (20–24 nt, S2 Table), indicating that the construct we prepared is as efficient at producing siRNAs as that reported previously [15]. While both 3- and 7-wag (weeks after grafting) lateral shoots of rootstock potato exhibited almost the same levels and characteristics in terms of siRNA profiles, the profile of epigenetic potato (Epi-A) showed only 0.07 reads-MR, being lower than that for WT potato (Fig 4 and S2 Table). These results suggest that the siRNAs are transported continuously as long as the graft remains established, but then are decompose quickly. Finally, to clarify whether or not methylation by the graft-transported siRNA occurs at sites other than the intended target, we analyzed off-target effects using our deep seq analysis data. Only one siRNA sequence (0.15 reads/million in the transgenic t33) out of 377 types of 24-nt siRNAs detected 54 putative off-target sites in the potato genome (*Solanum tuberosum*, PGSC V4.03). All of these sites comprised the 14-bp core sequence and a SSR (AAG / CTT), and adjacent sequences of about 10 bp were also homologous with each other (S11A Fig). The methylation levels of two out of 54 putative off-target sites were analyzed because both were located within the intron of a gene (function unknown) (S11B Fig). Both levels of methylation were not significantly different from that of the WT line (S11C Fig), indicating that both putative off-target sites were not modified at all. As the performance of RdDM requires cooperative work with RNA pol V products and DRM2 (DOMAINS REARRANGED METHYLTRANSFERASE 2), it is considered that the achievement is not merely because of the same sequence with the 24-nt siRNA.

Obtaining TGS MTs is very convenient for potato propagation because they can be used directly as seed potatoes. In the present study, however, all of the tubers formed from the hetero-graft plants did not manifest the TGS. Probably, when the callus formed on the cut surface of a lateral shoot is derived from distinct TGS cells of vascular bundle tissue, the whole TGS shoot and MT would be obtainable by stable maintenance of the epigenetic change through mitosis. Thus, it is considered that the use of a scion having a high degree of siRNA transport ability and the timing of callus formation on the cut surface are important for obtaining the TGS MTs.

In 2012, eight new plant breeding techniques (NPBT) were introduced [25]. Our graft-induced TGS involves a combination of two such technologies: grafting and RdDM (RNA-dependent DNA methylation). Grafting [26] as a NPBT involves culture of a chimeric plant formed between a non-GM scion on a GM root stock [27]. Although some specific RNAs and proteins can move through the graft union [28, 29], there is no evidence that the transgene moves into the graft partner [27]. With our technique, the scion is used as the supplier of a specifically designed siRNA molecule and the root stock provokes epigenetic changes through the natural system of RdDM that already exists in plant cells. When evaluating the resulting product from the viewpoint of NPBT [30], the potato thus created would be exempt from GM restriction. Furthermore, the epigenetically modified potato would not retain the functional siRNA, and the resulting plant would not contain any recombinant DNA. Large-scale cultivation of the epigenetically improved potato is now being planned to verify the stable maintenance of TGS, and another interesting gene in the potato genome is also being targeted for TGS. Thus, we have exploited an epigenome editing technology by mobile RNA silencing for crop improvement [31].

## Materials and Methods

### Plant materials and growth conditions

Potato (*Solanum tuberosum* tetraploid cultivar ‘Waseshiro’) shoots were grown *in vitro* on MS medium [32] with 30 g/l sucrose and 3 g/l Gellan gum (Wako Pure Chemical, Japan), at 24°C under a 16-h light / 8-h dark cycle with cool fluorescent light at about 100 μmol m^–2^ s^–1^.

### Generation of transgenic plants

A *mGFP5-ER gene* (accession no. U87974) from GFP transgenic *Nicotiana benthamiana* line 16C [33], amplified by PCR using primer GFP FP and RP, was inserted into the *Xba*I/*Sac*I sites of pIG121 [34]. mGFP5-ER nearly full length (670 bp out of 792 bp) amplified by PCR using primer GFP S5’FP and S3’RP, was inserted into the pBluescript SKII (+) (STRATAGENE) by TA cloning. The *CAT1* intron [34], amplified by PCR using primer intFP and intRP, was inserted into the *Aat*II/*Hin*dIII sites as a spacer. For the reverse sequence, an amplification product obtained using primers GFP A3’FP and A5’RP was inserted into the *Hin*dIII/ *Kpn*I sites. Finally, CoYMV:GFPIR was completed by inserting GFPIR into the *Bam*HI/*Kpn*I sites of CoYMV-NOSter [16]. The promoter sequence of *GBSSI* was obtained from the data of Sol genomic network data (http://solgenomics.net/) and a previous paper [23] by PCR using stGBSSIFP and RP. Transformation of potato and the formation of MT was performed essentially as described [18, 35], respectively. Transformation of tobacco and construction of the empty vector was performed as described previously [36]. DNA sequences of each plasmid were confirmed using an ABI 3500 Genetic Analyzer (Life Technologies). All of the primer sequences used in this study are listed in S1 Table.

### Methylation assays

Total genomic DNA was extracted in CTAB buffer (3% CTAB, 0.1 M Tris-HCl pH 8.0, 20 mM EDTA, 1.4 M NaCl) from 50 to 100 mg of plant tissue (leaves or MT). After treatment with RNaseA (Nacalai Tesque, Japan), gDNA was purified using a QuickGene DNA tissue Kit S and QuickGene-Mini80 (KURABO, Japan). The level of DNA methylation was analyzed by treatment with the methylation-dependent restriction enzyme *Mcr*BC and qRT-PCR. Bisulfite sequencing and the qPCR analysis were performed essentially as described [14] using a SsoFast EvaGreen Supermix with a Chrome4 real-time PCR detector (Bio-Rad). The primers used for the methylation assays are described in S1 Table.

### Total RNA extraction and qPCR analysis

Total RNA from leaves was extracted using TRizol reagent (Life Technologies). In the case of MT total RNA, the tuber was ground with a mortar and pestle using liquid N_2_, then the powder was suspended in 300 μl washing buffer (0.1 M Tris-HCl pH 8.0, 0.1% PVP, 4% 2-Me). The supernatant (250 μl) of the centrifuged mixture was treated with 750 μl Trizol LS reagent, and followed by protocols (Life Technologies). In brief, extracted total RNA was treated with TURBO DNA-free. cDNA was synthesized with 1 μg RNA as a template using a Superscript VlLO. The primers used for qPCR are described in S1 Table.

### Northern blot analysis

The initial step for extraction of small RNAs was performed essentially as described previously [36]. The small RNAs obtained were refined by filter purification using mirVana miRNA isolation kit (Life Technologies) in accordance with the manufacturer's instructions. Detection of siRNAs was performed as described previously [36]. The DIG-labeled sense and antisense riboprobe corresponding promoter regions sequence of the target was synthesized using the DIG RNA Labeling kit (Roche).

### Southern blot analysis

Genomic DNA of transformants was extracted from 0.5 to 2.0 g of leaves using the Urea buffer. Fifteen micrograms of genomic DNA was digested with a restriction enzyme and separated by electrophoresis on a 1% agarose gel. The electrophoresed DNA was then blotted onto a nylon membrane (Pall Corp.). Digoxigenin-labeled DNA probes corresponding to 35S promoter were synthesized using a DIG DNA labeling kit (Roche). The DIG DNA probe was hybridized to the DNA at 45°C in DIG Easy Hyb solution. The membrane was washed twice with 2×SSC/0.1% SDS at room temperature and then twice with 0.5×SSC/0.1% SDS at 68°C. Visualization and detection were performed in the same way as that for Northern blot analysis.

### Micrografting, regeneration from roots, and MT formation

The siRNAs donor *N*. *benthamiana* plants were germinated on MS medium [32] and the siRNA recipient *S*. *tuberosum* shoots were from plant subcultured on MS30 medium. A shoot of *N*. *benthamiana* was cut horizontally at approximately 2~3 cm below the tip and its cut side was inserted into a silicone tube (5 mm length, 1 mm internal × 2 mm external diameter) with a vertical slit. Then, the siRNAs recipient potato was also cut horizontally at approximately 2~3 cm above the rooting part including at least one lateral bud and was also inserted into the silicone tube to allow both cut surfaces to cohere. The shoot that sprouted from the lateral bud of the potato stock was cut off using a razor, and the hetero-grafted plants were grown for a further two months. Then, the roots were harvested to analyze their methylation and to obtain the regenerated shoot by culture on MS20, BAP 3 mg/l, TDZ 0.1 mg/l, agar 7 g/l for two weeks, and then on MS20, zeatin 3 mg/l, IAA 10 μg/l, GA_3_ 0.2 mg/l, Gellan gum 3 g/l for further two weeks, To obtain MT, the shoots were placed on MS80 containing 5 μM BAP [18].

### GFP imaging

GFP fluorescence was photographed using a digital camera (α58, SONY) with a UV-cut filter (Y2 Professional, Kenko·Tokina, Japan) under UV light from a hand-held 100 W long-wavelength UV lamp (B100AP; UVP Ultraviolet Products, Upland, USA). GFP fluorescence on MT samples was monitored with a biological fluorescence microscope (BX61, Olympus, Japan), and their digital images were captured with a digital camera (DP71, Olympus) connected to the microscope.

### Amylose content

The amylose percentage was determined spectrophotometrically in 100 mg of extracted starch according to the method described previously [37].

### Extraction of small RNAs and detection of siRNAs

The initial step for extraction of small RNAs was performed essentially as described previously [36]. The small RNAs obtained were refined by filter purification using a mirVana miRNA isolation kit (Life Technologies) in accordance with manufacturer’s instructions. Detection of siRNAs was performed essentially as described previously [14]. The DIG-labeled sense and antisense riboprobe corresponding the promoter region sequence of the target was synthesized using the DIG RNA Labeling kit (Roche).

### Next-generation sequencing

Twenty micrograms of total RNA was extracted from shoots of *in vitro* cultured WT, epigenetically changed potato (Epi-A), t33 and lateral shoots of hetero-grafted plants. Respective samples were composed of at least 15 individuals. Extracted RNA samples were sent to Hokkaido System Science (Sapporo, Japan) for next-generation sequence analysis using an Illumina HiSeq (Illumina, San Diego, CA). After removing the adapter sequences, non-redundant reads (>16 nt) were retained. The filtered reads were analyzed to match against the *StGBSS* 5' region a or b (S6 Fig), respectively. The resulting reads are summarized in S2 Table.

## Supporting Information

S1 Fig Regeneration from the callus formed on cut surface of potato shoot. After cutting by a razor blade (0 dac, red arrow head), small callus was formed on the surface (11 dac) and then adventitious bud (15 dac) and the shoot (21 dac) grew. To observe clearly the adventitious shoot formation, subcultured shoot was used alone.
(TIF)

S2 Fig Maintenance of TGS status in the progenies. (**A**) Methylation level in the target region. Second progeny tubers of the 35S:GFP line (35SGFP), WT/35S:GFP (Cont), CoGFPIR/35S:GFP (PTGS), and Co35SIR/35S:GFP (TGS). (**B**) GFP expression in the 2nd progeny tubers. The MTs were cut with a razor blade and their surface was observed under UV light. (**C**) Level of the GFP transcript. Asterisks show statistically significant (*; p<0.05 Student's t test) differences relative to 35SGFP. Means and SD of 3 biological replicates are shown.
(TIF)

S3 Fig TGS induction in MTs on potato rootstock grafted with Co35SIR *N*. *benthamiana* scion. (A) MT formed on a regenerated lateral shoot of root stock potato. (B) GFP expression of MTs. (C) Methylation level of the target region. (D) Methylation level of non-target region (+113~+782 bp of GFP). Asterisks show statistically significant (*; p<0.05 Student's t test) differences relative to 35SGFP. Error bars indicate 95% confidence intervals from 2 to 4 biological replicates.
(TIF)

S4 Fig TGS manifestation of 35S:GFP in roots of 35SGFP potato grafted with Co35SIR *N*. *benthamiana* scion. (A) The roots harvested at two months after grafting. Top shows bright-field images and bottom shows UV fluorescence images. (B) Methylation levels in bulked roots. (C) Levels of GFP transcript in bulked root. Asterisks show statistically significant (*; p<0.05 Student's t test) differences relative to 35SGFP. Means and SD of 3 biological replicates are shown.
(TIF)

S5 Fig TGS of 35S:GFP in regenerated shoot from the root of the grafted plant between Co35SIR *N*. *benthamiana* scion and potato rootstock. (**A**) Regenerated potato shoots and their GFP expressions. (**B**) Methylation levels of target and non-target (+113 ~ +782 bp of GFP) regions in regenerated shoots. (**C**) GFP expression of MTs formed on the regenerated shoots. Asterisks show statistically significant (*; p<0.05 Student's t test) differences relative to 35SGFP. Error bars indicate 95% confidence intervals from 2 to 4 biological replicates.
(TIF)

S6 Fig *GBSSI-a* and *GBSSI-b* of potato cultivar 'Waseshiro'. (**A**) Schematic presentation of the difference between the 5’flanking regions of a and b allele. (**B**) Sequence alignment of the 5’flanking regions of a and b allele.
(TIF)

S7 Fig Characterization of CoGBpIR transgenic potato line t33. (**A**) Southern hybridization. (**B**) Northern blot analysis for siRNA of target region.
(TIF)

S8 Fig Northern blot analysis of siRNAs targeting the *GBSSI* promoter in the transgenic *N*. *benthamiana*. Small RNA enriched nucleic acid (10 μg) was analyzed in 15% polyacrylamide gel and probed with the promoter negative (top) and positive (middle) strand RNA. 5.8S rRNA hybridization (bottom) was used as a loading control.
(TIF)

S9 Fig Genomic PCR of *GBSSI* Epi-A potato. (**A**) Schematic diagrams of the TGS starter CoGBpIR. Orange line indicates the target region. Blue bars (1~7) show the regions where PCR experiments were carried out to know whether the starter CoGBpIR is present in the potato. (**B**) Genomic PCR products in each region. From left to right, WT potato, A1 and A2 lines of Epi-A potato, and CoGBpIR *N*. *benthamiana*. *Actin* gene of *S*. *tuberosum* and *ubiquitin* gene of *N*.*b*. are amplified as controls. The size (bp) of the PCR products are shown at the both sides. (**C**) Results arranged in each PCR experiment. In region 5, two alleles of *GBSSI-a* and *-b* (Fig 3) were amplified. (**D**) Cycling conditions of the PCR.
(TIF)

S10 Fig Methylation level of the *GBSSI* target region in a shoot from the root of a hetero-grafted CoGBpIR *N*. *benthamiana* scion and potato rootstock. Three independent TGS lines regenerated from the grafted plant roots were analyzed. Asterisks show statistically significant (*; p<0.05, **; P<0.01 Student's t test) differences relative to the WT. Means and SD of 3 biological replicates are shown.
(TIF)

S11 Fig Analysis of putative off-target effects on a *GBSSI* TGS line. (A) Sequence alignment of putative off-target sites on PGSC (Potato Genome Sequencing Consortium) data. Each site is indicated with the chromosome number and start position. Two sequences analyzed for their methylation level are shown against a yellow background. Off-target sequences are shown in red and blue, and black underlining indicates the core sequence. (B) Sequence alignment of two off-target sites between PGSC and ‘Waseshiro’ WT. SSR = simple sequence repeat. (C) Methylation level of the *GBSSI* target region and two off-target sites. Asterisks show statistically significant (**; P<0.01 Student's t test) differences relative to the WT. Means and SD of 3 biological replicates are shown.
(TIF)

S1 Table List of oligonucleotides used in this study. (DOCX)

S2 Table Summary of siRNAs mapped to the target region of *GBSSI*. (DOCX)
