# Supplementary figures and images for: Epigenome Editing of Potato by Grafting Using Transgenic Tobacco as siRNA Donor
Source: PLoS One. 2016 Aug 26;11(8):e0161729. doi: 10.1371/journal.pone.0161729 (PMC5001710; doi:10.1371/journal.pone.0161729)

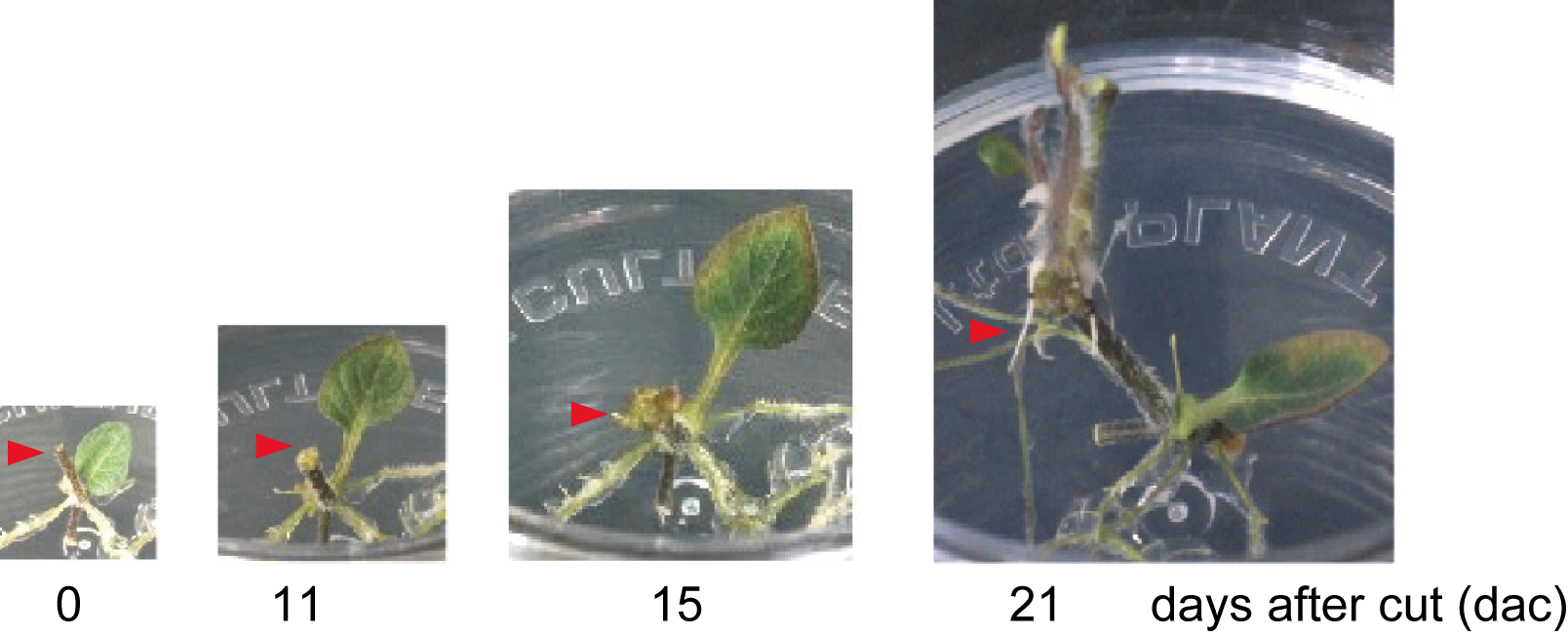

Supplement: S1 Fig — After cutting by a razor blade (0 dac, red arrow head), small callus was formed on the surface (11 dac) and then adventitious bud (15 dac) and the shoot (21 dac) grew. To observe clearly the adventitious shoot formation, subcultured shoot was used alone. (TIF) [file pone.0161729.s001.tif]

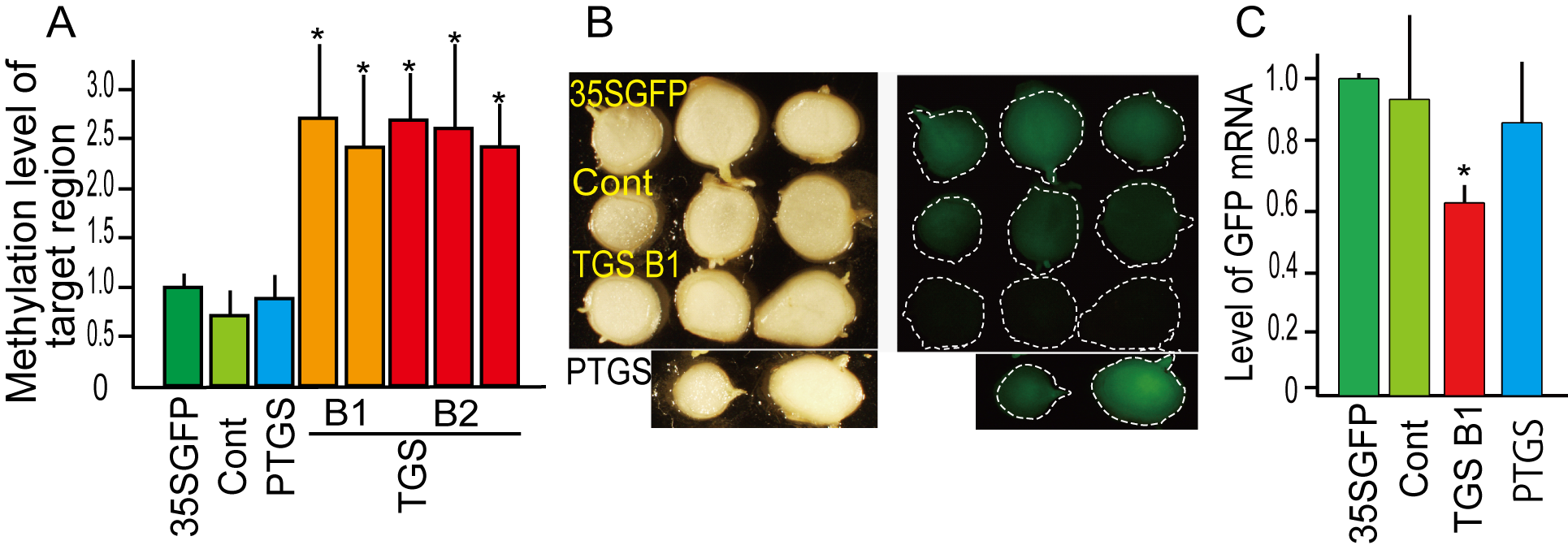

Supplement: S2 Fig — (A) Methylation level in the target region. Second progeny tubers of the 35S:GFP line (35SGFP), WT/35S:GFP (Cont), CoGFPIR/35S:GFP (PTGS), and Co35SIR/35S:GFP (TGS). (B) GFP expression in the 2nd progeny tubers. The MTs were cut with a razor blade and their surface was observed under UV light. (C) Level of the GFP transcript. Asterisks show statistically significant (*; p<0.05 Student's t test) differences relative to 35SGFP. Means and SD of 3 biological replicates are shown. (TIF) [file pone.0161729.s002.tif]

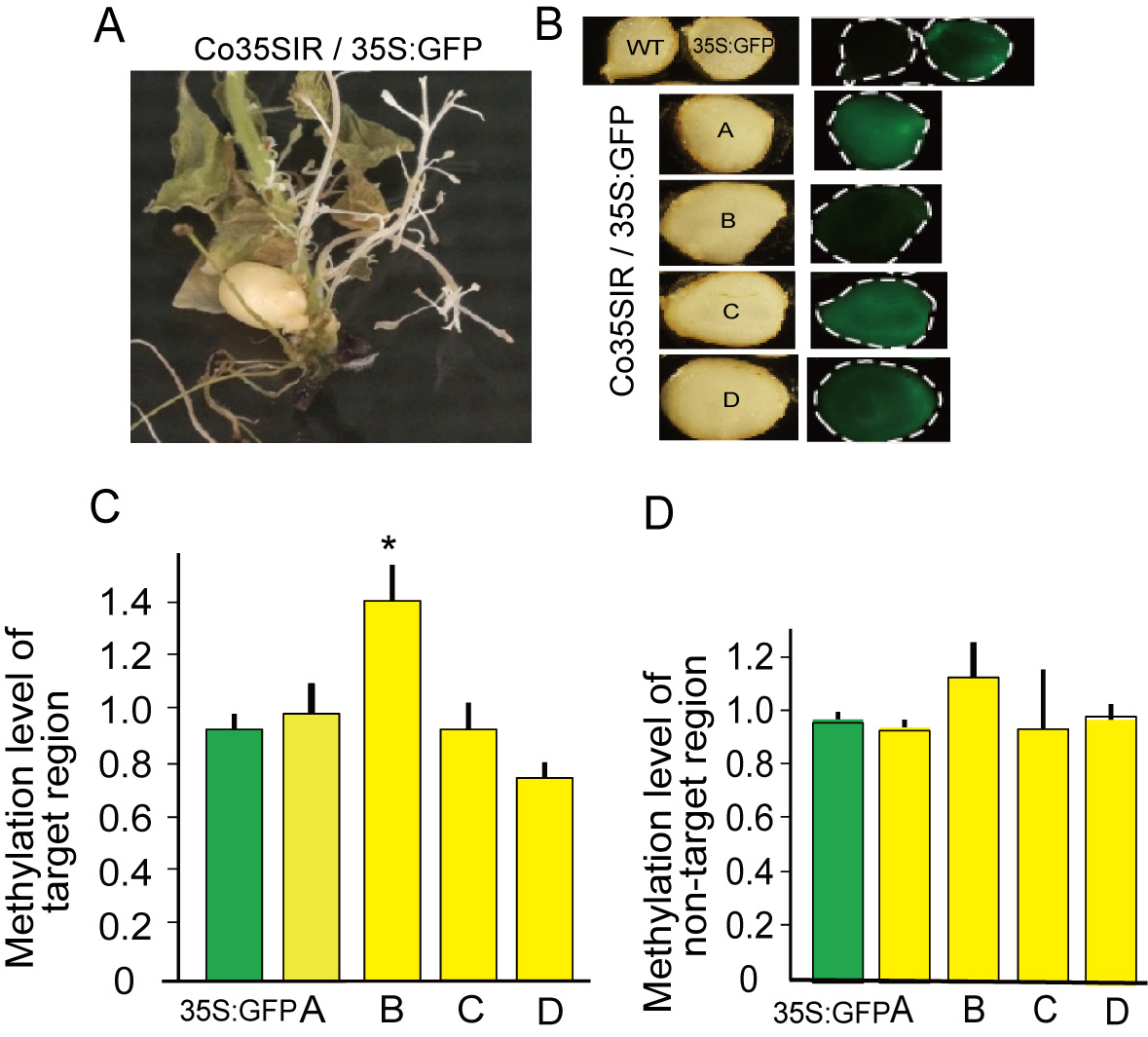

Supplement: S3 Fig — (A) MT formed on a regenerated lateral shoot of root stock potato. (B) GFP expression of MTs. (C) Methylation level of the target region. (D) Methylation level of non-target region (+113~+782 bp of GFP). Asterisks show statistically significant (*; p<0.05 Student's t test) differences relative to 35SGFP. Error bars indicate 95% confidence intervals from 2 to 4 biological replicates. (TIF) [file pone.0161729.s003.tif]

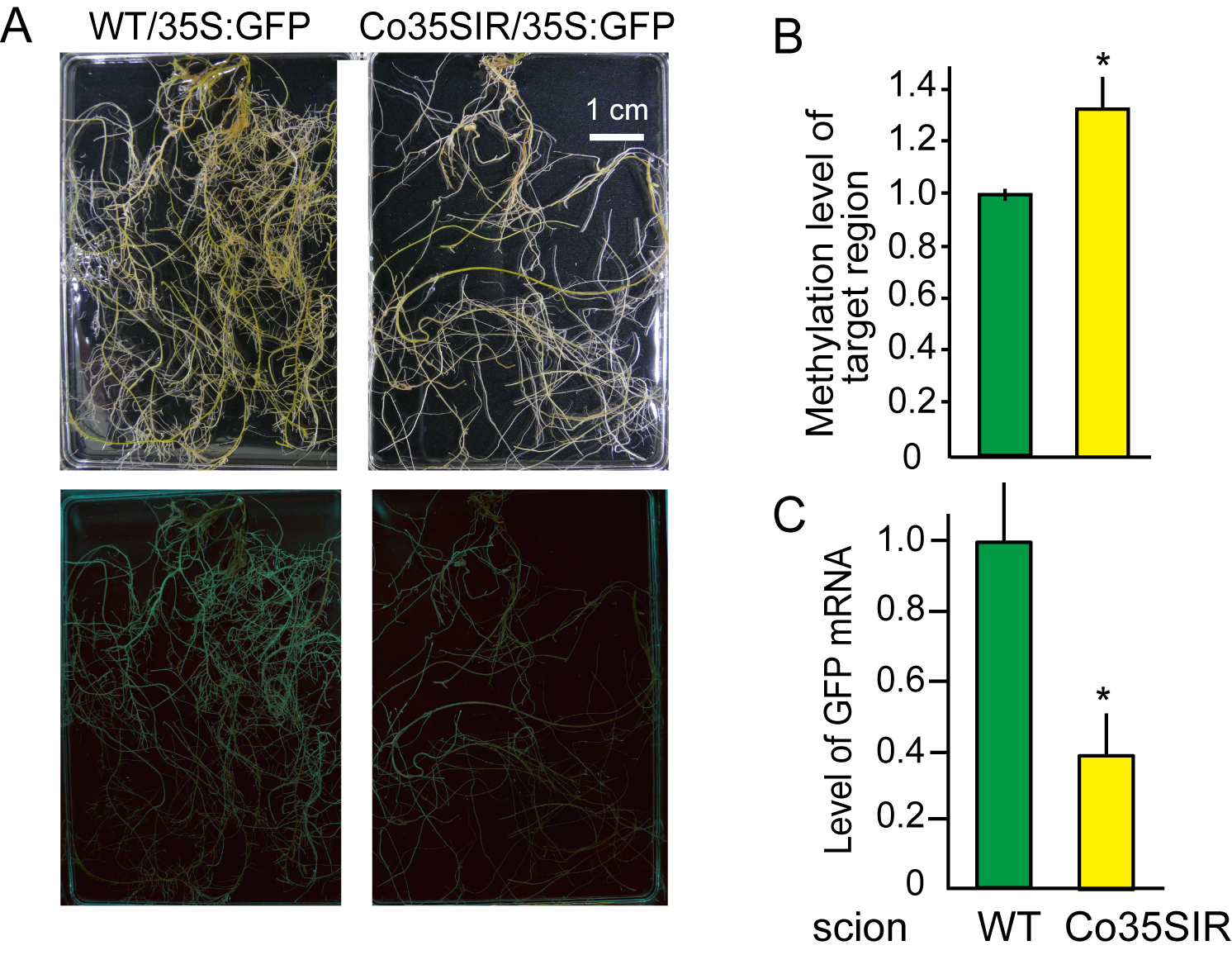

Supplement: S4 Fig — (A) The roots harvested at two months after grafting. Top shows bright-field images and bottom shows UV fluorescence images. (B) Methylation levels in bulked roots. (C) Levels of GFP transcript in bulked root. Asterisks show statistically significant (*; p<0.05 Student's t test) differences relative to 35SGFP. Means and SD of 3 biological replicates are shown. (TIF) [file pone.0161729.s004.tif]

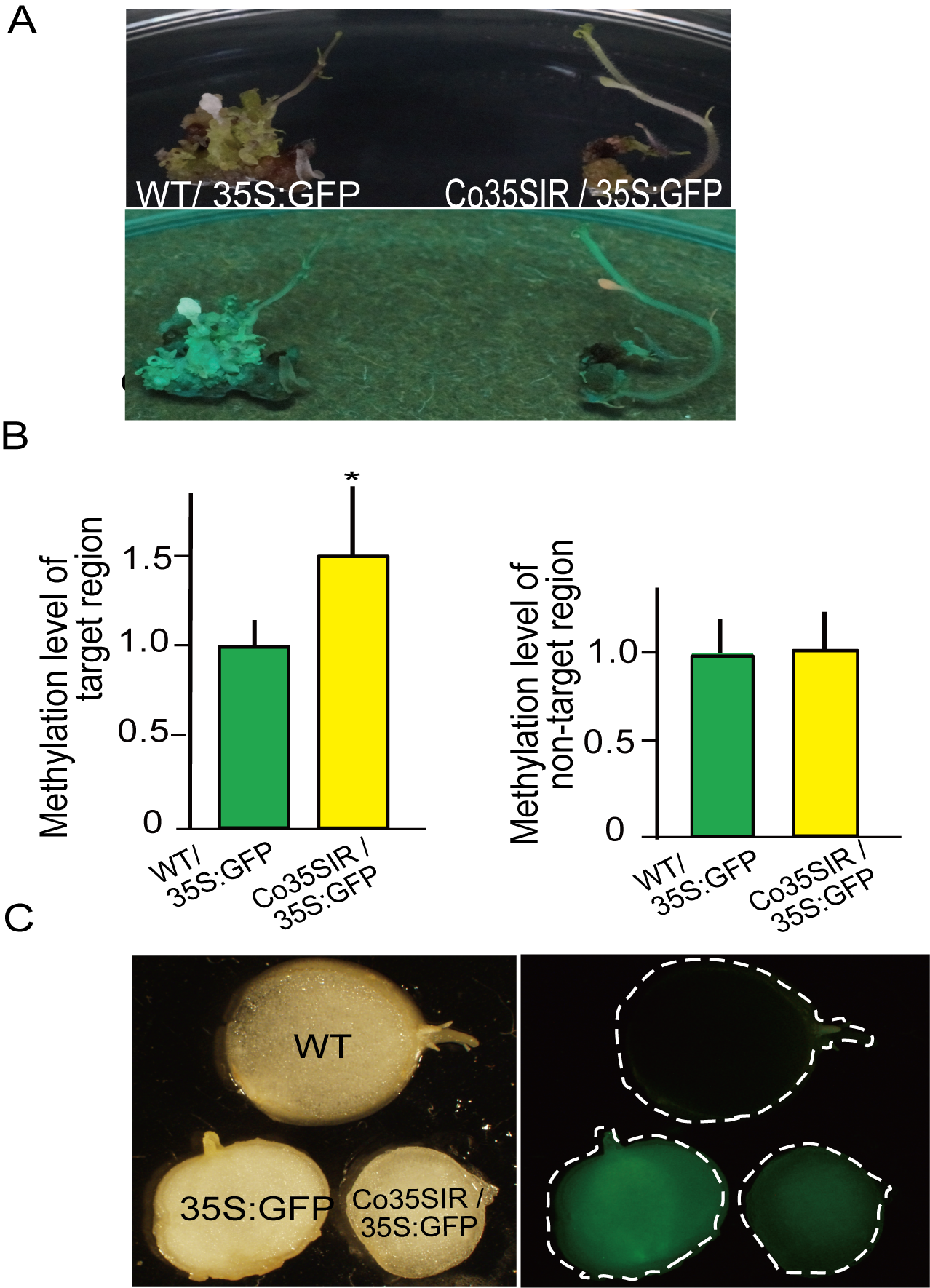

Supplement: S5 Fig — (A) Regenerated potato shoots and their GFP expressions. (B) Methylation levels of target and non-target (+113 ~ +782 bp of GFP) regions in regenerated shoots. (C) GFP expression of MTs formed on the regenerated shoots. Asterisks show statistically significant (*; p<0.05 Student's t test) differences relative to 35SGFP. Error bars indicate 95% confidence intervals from 2 to 4 biological replicates. (TIF) [file pone.0161729.s005.tif]

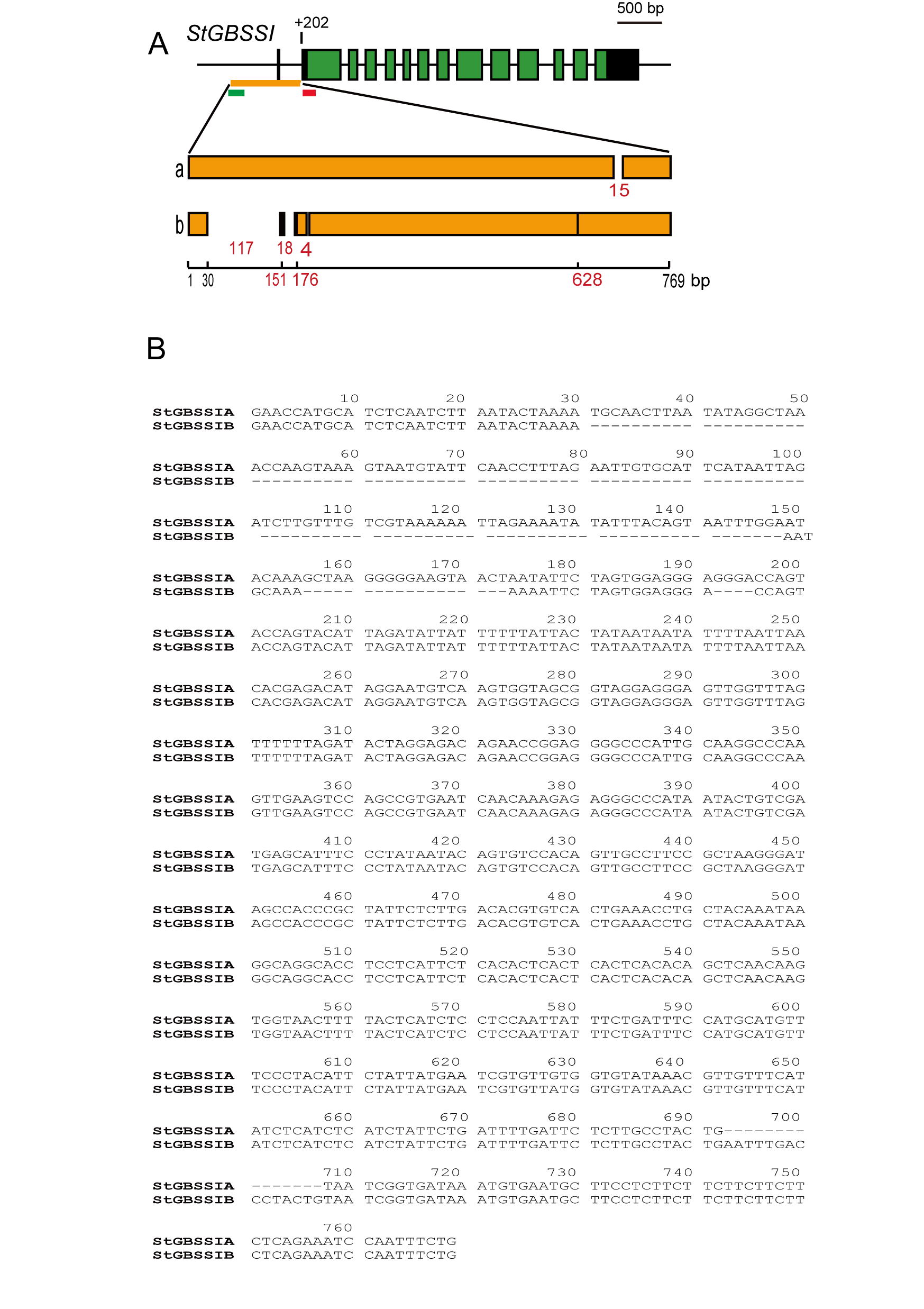

Supplement: S6 Fig — (A) Schematic presentation of the difference between the 5’flanking regions of a and b allele. (B) Sequence alignment of the 5’flanking regions of a and b allele. (TIF) [file pone.0161729.s006.tif]

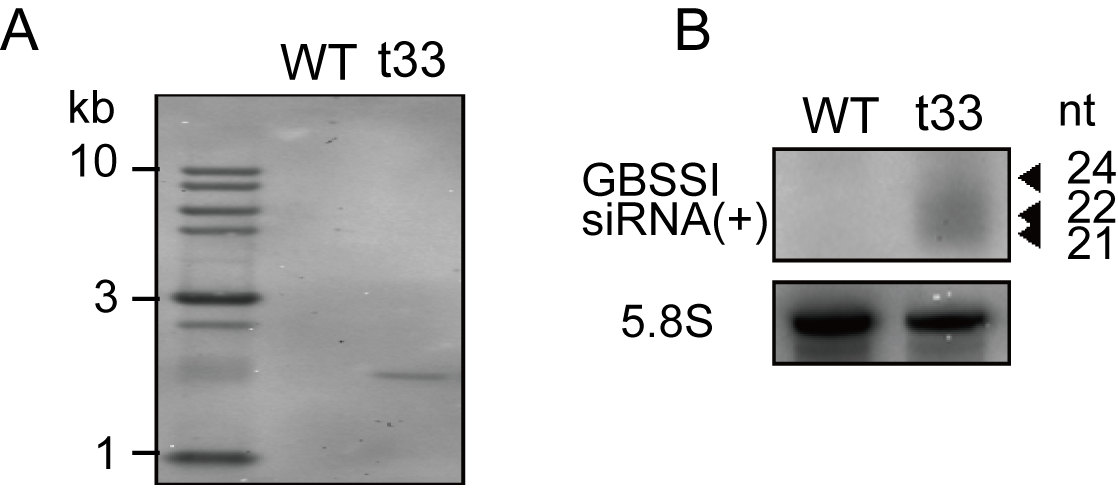

Supplement: S7 Fig — (A) Southern hybridization. (B) Northern blot analysis for siRNA of target region. (TIF) [file pone.0161729.s007.tif]

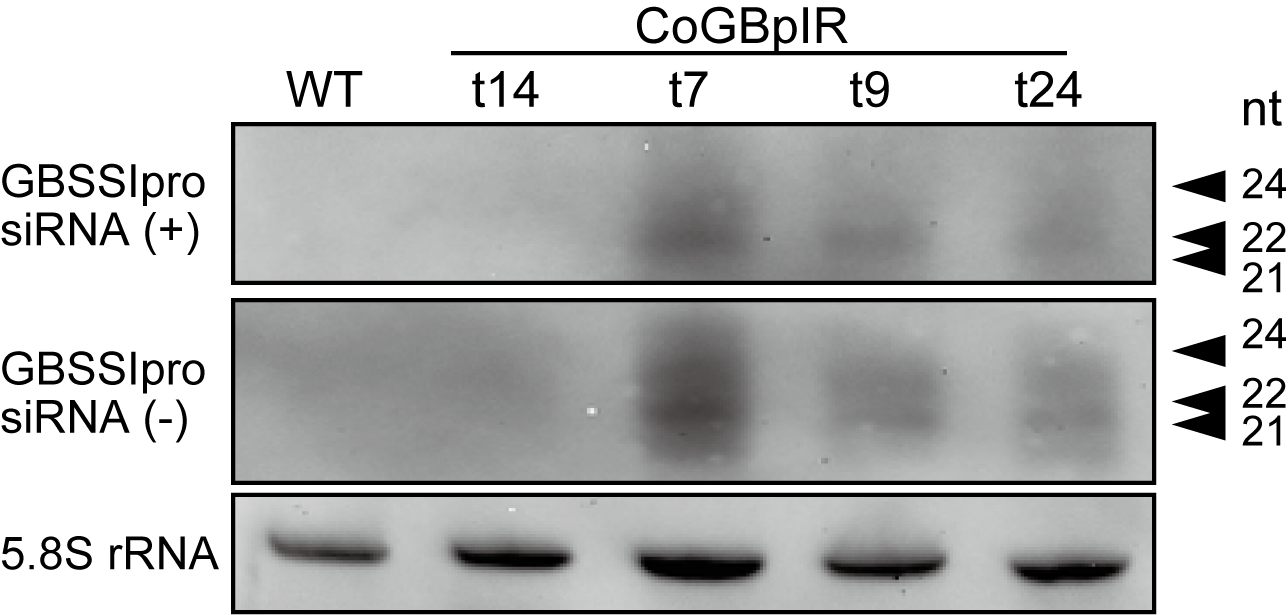

Supplement: S8 Fig — Small RNA enriched nucleic acid (10 μg) was analyzed in 15% polyacrylamide gel and probed with the promoter negative (top) and positive (middle) strand RNA. 5.8S rRNA hybridization (bottom) was used as a loading control. (TIF) [file pone.0161729.s008.tif]

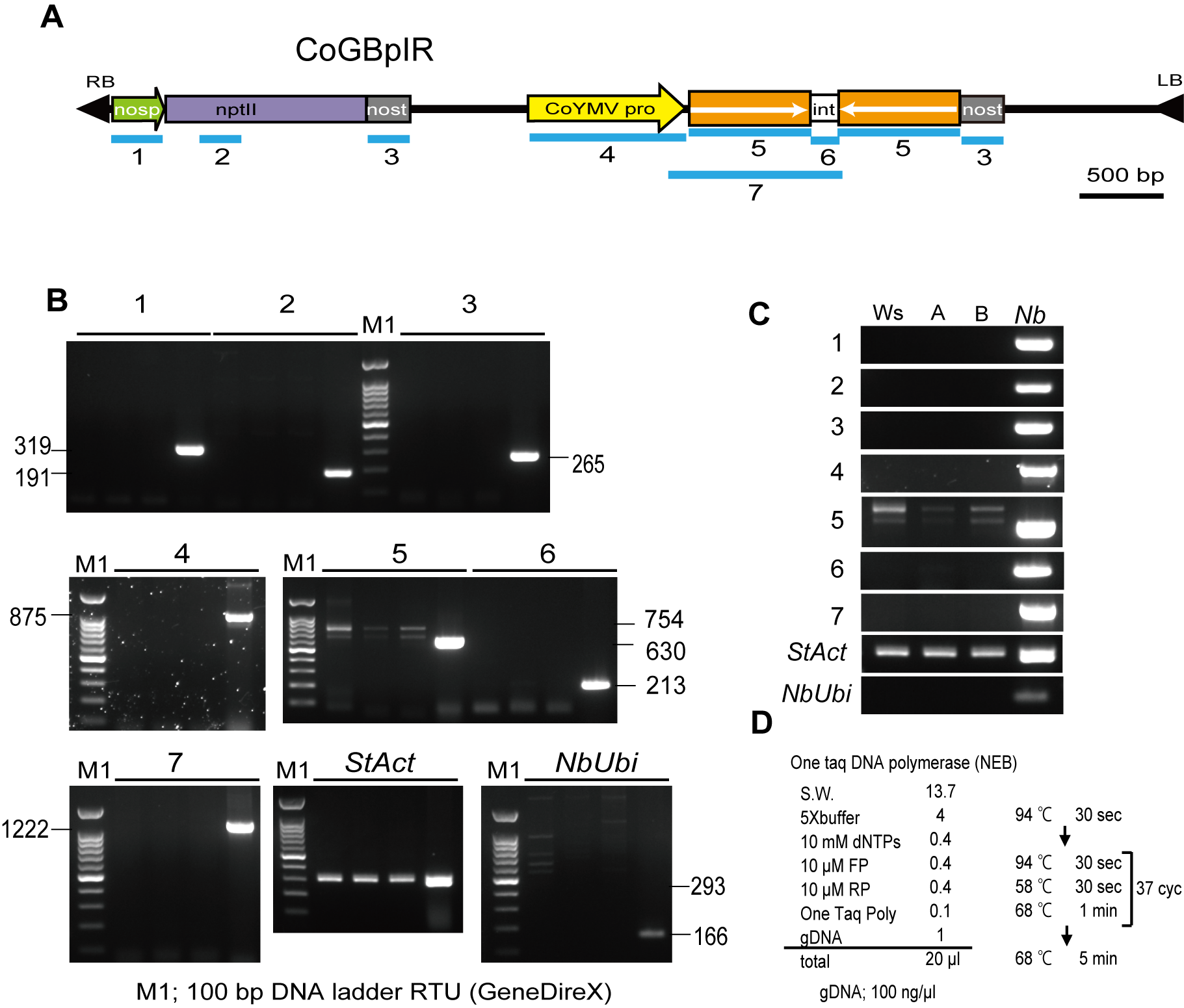

Supplement: S9 Fig — (A) Schematic diagrams of the TGS starter CoGBpIR. Orange line indicates the target region. Blue bars (1~7) show the regions where PCR experiments were carried out to know whether the starter CoGBpIR is present in the potato. (B) Genomic PCR products in each region. From left to right, WT potato, A1 and A2 lines of Epi-A potato, and CoGBpIR N. benthamiana. Actin gene of S. tuberosum and ubiquitin gene of N.b. are amplified as controls. The size (bp) of the PCR products are shown at the both sides. (C) Results arranged in each PCR experiment. In region 5, two alleles of GBSSI-a and -b (Fig 3) were amplified. (D) Cycling conditions of the PCR. (TIF) [file pone.0161729.s009.tif]

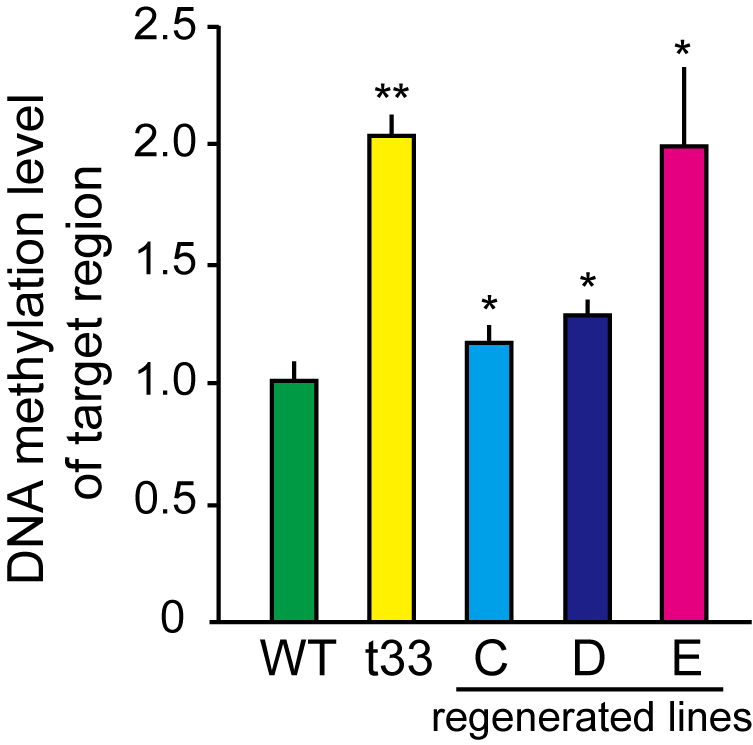

Supplement: S10 Fig — Three independent TGS lines regenerated from the grafted plant roots were analyzed. Asterisks show statistically significant (*; p<0.05, **; P<0.01 Student's t test) differences relative to the WT. Means and SD of 3 biological replicates are shown. (TIF) [file pone.0161729.s010.tif]

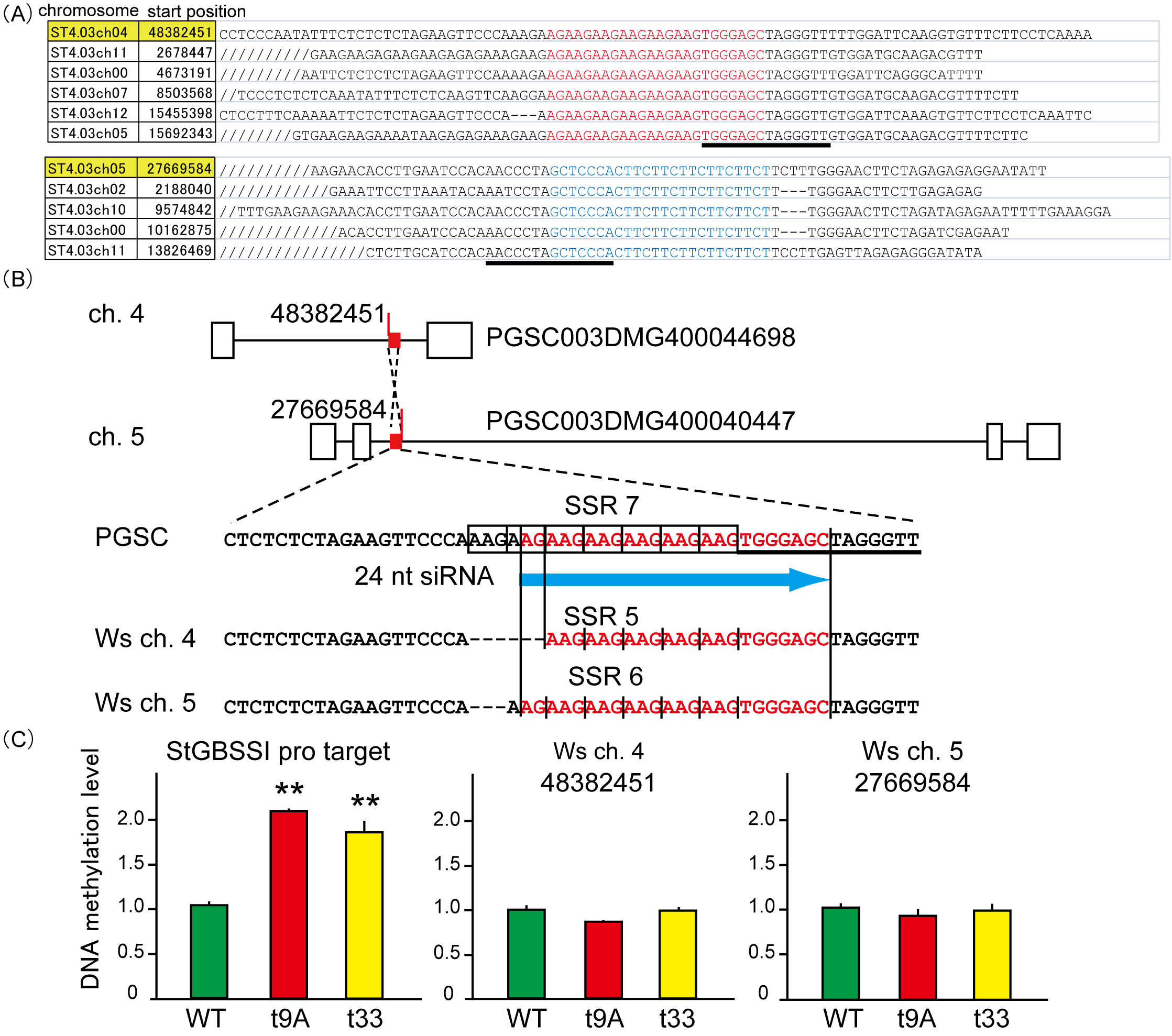

Supplement: S11 Fig — (A) Sequence alignment of putative off-target sites on PGSC (Potato Genome Sequencing Consortium) data. Each site is indicated with the chromosome number and start position. Two sequences analyzed for their methylation level are shown against a yellow background. Off-target sequences are shown in red and blue, and black underlining indicates the core sequence. (B) Sequence alignment of two off-target sites between PGSC and ‘Waseshiro’ WT. SSR = simple sequence repeat. (C) Methylation level of the GBSSI target region and two off-target sites. Asterisks show statistically significant (**; P<0.01 Student's t test) differences relative to the WT. Means and SD of 3 biological replicates are shown. (TIF) [file pone.0161729.s011.tif]
