# Supplementary material for: Epigenome Editing of Potato by Grafting Using Transgenic Tobacco as siRNA Donor
Source: PLoS One. 2016 Aug 26;11(8):e0161729. doi: 10.1371/journal.pone.0161729 (PMC5001710; doi:10.1371/journal.pone.0161729)
Supplement: S1 Table — (DOCX) [file pone.0161729.s012.docx]

| **Oligonucleotide** | **Sequence(5' to 3')** | **RE site** |
| --- | --- | --- |
| **construction** |  |  |
| GFP FP  GFP RP | CTTCTAGAAAGGAGATATAACAATGAAG  CTGAGCTCCTTAAAGCTCATCATGTTTGT | *Xba*I  *Sac*I |
| GFP S5'FP  GFP S3'RP | GAGGATCCAGTTGTCCCAATTCTTGTTG  GCGACGTCCATGCCATGTGTAATCCCAGC | *Bam*HI  *Aat*II |
| int FPAa  int RPH | GCGACGTCCTACAGGGTAAATTCTAGTTTTTC  GCAAGCTTGGTTCTGTAACTATCATCATCATC | *Aat*II  *Hin*dIII |
| GFP A3'FP  GFP A5'RP | GCAAGCTTCATGCCATGTGTAATCCCAGC  GCGGTACCAGTTGTCCCAATTCTTGTTG | *Hin*dIII  *Kpn*I |
| StGBSSI FP  StGBSI RP | TTCTCGAGGCAGTAAAGGTTCCAC  GCTTGCCATGTGATGTGTGGTCTAC |  |
| StGBSS1prosFPB  StGBSS1prosRPA  StGBSS1proaFPH  StGBSS1proaRPK | GAGGATCCGAACCATGCATCTCAATCTT  GCGACGTCCAGAAATTGATTTCTGAGAAG  GCAAGCTTCAGAAATTGATTTCTGAGAAG  GCGGTACCGAACCATGCATCTCAATCTT | *Bam*HI  *Aat*II  *Hin*dIII  *Kpn*I |
| **methylaiton** |  |  |
| 35Spro RT FP  35Spro RT RP | GGTAATATCCGGAAACCTCCTC  CATCTTCAACGATGGCCTTTCC |  |
| mGFP FP  mGFP RP | GAGGGATACGTGCAGGAGAG  CGGCCATGATGTATACGTTG |  |
| mGFP FP  mGFP RP | GAGGGATACGTGCAGGAGAG  CGGCCATGATGTATACGTTG |  |
| StGBSS15proLeP  StGBSS15proRiP | GTTGTTTTTAACCATCCTTCC  ATCTAATGTACTGGTACTGGTCC |  |
| StGBSS13proLeP  StGBSS13proRiP  StAct1 gLeP  StAct1 gRiP | CTGATTTTGATTCTCTTGCCTACTG  CTTGCCATGTGATGTGTGGT  TGACCACTTTCCGATCTCCT  TAATCCCCACATGCATCTCA |  |
| qRT-PCR |  |  |
| StGBSS1LeP  StGBSS1RiP | GTAGATTCCCCTTTTTGTAGACC  CAGCCCTTAAACCATTGTGA |  |
| StActinF  StActinR | TCCCAAGGCCAACAGAG  GCAAGGTCCAAACGAAGA |  |
| **Bisulfite sequence** |  |  |
| StGBSS15proBS2LeP  StGBSS15proBS2RiP | TTYTYGAGGYAGTAAAGGTT  CAATRTTTRTTACATTTCTTCCTAT |  |
| StGBSS15proBSLeP  StGBSS15proBSRiP | TAGYAGTGTATYAATTTTGTAATAG  CCCTCTCTTTRTTRATTCAC |  |
| StGBSS13proBSLeP  StGBSS13proBSRiP | GGTTTAGTTTTTTAGATAYTAGGAG  TTAACARCCCTTAAACCATT |  |
| StGBSS13proBS2LeP  StGBSS13proBS2RiP | AAGGGYTGTTAAYAAGYTTGATGGG  CTCAACCRCAACRCTARTATCCCAA |  |

S1 Table. List of oligonucleotides used in this study.
