# Supplementary material for: Epigenome Editing of Potato by Grafting Using Transgenic Tobacco as siRNA Donor
Source: PLoS One. 2016 Aug 26;11(8):e0161729. doi: 10.1371/journal.pone.0161729 (PMC5001710; doi:10.1371/journal.pone.0161729)
Supplement: S2 Table — (DOCX) [file pone.0161729.s013.docx]

S2 Table. Summary of siRNAs mapped to the target region of *GBSSI.*

|  | 35S:GBSSpIR  t33 | Co:GBSSpBIR-Nb/Ws* 3 wag† | Co:GBSSpBIR-Nb/Ws 7 wag | *GBSSI* Epi-Ws | WT-Ws |
| --- | --- | --- | --- | --- | --- |
| Total reads | 13,628,750 | 11,650,432 | 12,599,867 | 14,208,462 | 11,856,368 |
| 20 nt (R/M‡) | 67.14 | 4.46 | 3.97 | 0.00 | 0.17 |
| 21 nt (R/M) | 1552.09 | 30.30 | 32.22 | 0.07 | 0.00 |
| 22 nt (R/M) | 382.79 | 74.93 | 59.92 | 0.00 | 0.00 |
| 23 nt (R/M) | 35.07 | 2.92 | 3.17 | 0.00 | 0.08 |
| 24 nt (R/M) | 132.81 | 14.16 | 12.30 | 0.00 | 0.17 |
| Total (R/M) | 2169.90 | 126.78 | 111.59 | 0.07 | 0.34 |

*Potato cultivar 'Waseshiro'. Underline means the material used for analyzing.

†Weeks after grafting.

‡Numbers in the same row indicate the mapped reads per million sequencing reads (20-24 nt)
